# Segregating the effects of ferric citrate‐mediated iron utilization and FGF23 in a mouse model of CKD

**DOI:** 10.14814/phy2.15307

**Published:** 2022-06-03

**Authors:** Michael P. Liesen, Megan L. Noonan, Pu Ni, Rafiou Agoro, Julia M. Hum, Erica L. Clinkenbeard, John G. Damrath, Joseph M. Wallace, Elizabeth A. Swallow, Matthew R. Allen, Kenneth E. White

**Affiliations:** ^1^ Department of Medical & Molecular Genetics Indiana University School of Medicine Indianapolis Indiana USA; ^2^ Department of Physiology Marian University Indianapolis Indiana USA; ^3^ Purdue University Weldon School of Biomedical Engineering West Lafayette Indiana USA; ^4^ Department of Biomedical Engineering Indiana University‐Purdue University at Indianapolis Indianapolis Indiana USA; ^5^ Department of Anatomy, Cell Biology, and Physiology Indiana University School of Medicine Indianapolis Indiana USA; ^6^ Department of Medicine Division of Nephrology Indiana University School of Medicine Indianapolis Indiana USA

**Keywords:** CKD, ferric citrate, GF23, iron, kidney, klotho

## Abstract

Ferric citrate (FC) is an approved therapy for chronic kidney disease (CKD) patients as a phosphate (Pi) binder for dialysis‐dependent CKD, and for iron deficiency anemia (IDA) in non‐dialysis CKD. Elevated Pi and IDA both lead to increased FGF23, however, the roles of iron and FGF23 during CKD remain unclear. To this end, iron and Pi metabolism were tested in a mouse model of CKD (0.2% adenine) ± 0.5% FC for 6 weeks, with and without osteocyte deletion of *Fgf23* (flox‐Fgf23/Dmp1‐Cre). Intact FGF23 (iFGF23) increased in all CKD mice but was lower in Cre^+^ mice with or without FC, thus the Dmp1‐Cre effectively reduced FGF23. Cre^+^ mice fed AD‐only had higher serum Pi than Cre^−^ pre‐ and post‐diet, and the Cre^+^ mice had higher BUN regardless of FC treatment. Total serum iron was higher in all mice receiving FC, and liver Tfrc, Bmp6, and hepcidin mRNAs were increased regardless of genotype; liver IL‐6 showed decreased mRNA in FC‐fed mice. The renal 1,25‐dihydroxyvitamin D (1,25D) anabolic enzyme Cyp27b1 had higher mRNA and the catabolic Cyp24a1 showed lower mRNA in FC‐fed mice. Finally, mice with loss of FGF23 had higher bone cortical porosity, whereas Raman spectroscopy showed no changes in matrix mineral parameters. Thus, FC‐ and FGF23‐dependent and ‐independent actions were identified in CKD; loss of FGF23 was associated with higher serum Pi and BUN, demonstrating that FGF23 was protective of mineral metabolism. In contrast, FC maintained serum iron and corrected inflammation mediators, potentially providing ancillary benefit.

## Graphical abstract

Chronic kidney disease (CKD) affects 10% of the world population and results in many patient complications, including iron deficiency and hyperphosphatemia, both potent stimulators of the phosphaturic hormone FGF23. An iron‐containing phosphate binder, ferric citrate (FC), was developed to alleviate these manifestations, however, the impact of FC on FGF23‐ and iron‐mediated effects in CKD are not completely understood. This study provides novel insight into the FGF23‐ and iron‐mediated effects in CKD. We demonstrate that FGF23 is necessary to control phosphate and disease progression (monitored by BUN) in CKD. This study also supports that FC may improve 1,25D metabolism independent of FGF23 and lower markers of inflammation due to iron repletion. Therefore, FC may provide ancillary benefits which could lead to improved CKD patient outcomes.

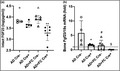

## 1 INTRODUCTION

Chronic kidney disease (CKD) is a global health concern (Ganz et al., 2019), and the underlying pathologic mechanisms causing the endocrine dysfunction in CKD remain unclear. FGF23 is a hormone produced principally in bone (Martin et al., 2012). To initiate bioactivity under conditions of normal renal function, FGF23 binds to the FGF receptor (FGFR) and its co‐receptor alpha‐Klotho (KL), which is primarily expressed in the kidney and parathyroid glands (Martin et al., 2012). In the kidney, FGF23 controls 1,25(OH)_2_ vitamin D (1,25D) metabolism and phosphate handling (Clinkenbeard & White, 2016; Martin et al., 2012). FGF23 acts as a counter‐regulatory hormone to elevated 1,25D (Martin et al., 2012) through suppressing the vitamin D 1α‐hydroxylase (Cyp27b1), an anabolic enzyme for 1,25D production, and in parallel increasing expression of the vitamin D 24‐hydroxylase (Cyp24a1), catabolic for 1,25D (Clinkenbeard et al., 2014, 2016; Faul et al., 2011). These combined actions protect from high levels of 1,25D and decrease intestinal phosphate absorption. FGF23 is also vital for coordinating renal phosphate flux by inhibiting kidney phosphate reabsorption by down‐regulating the proximal tubule sodium phosphate cotransporters NPT2a and NPT2c (Clinkenbeard et al., 2016; Farrow et al., 2010; Larsson et al., 2004; Martin et al., 2012).

In diseases of FGF23 over production where renal function is maintained, such as X‐linked hypophosphatemia (XLH) and autosomal dominant hypophosphatemic rickets (ADHR), high levels of FGF23 reduce 1,25D production and cause hypophosphatemia, impaired growth, rickets, and osteomalacia (Farrow et al., 2011; Ganz et al., 2019; Martin et al., 2012). During CKD, kidney KL expression gradually declines (Hu et al., 2011), causing progressive, compensatory FGF23 increases in patients in an attempt to maintain physiological blood phosphate concentrations. At the late stages of CKD, FGF23 can reach over 1000 times normal concentrations (Isakova, 2011). Hyperphosphatemia in CKD, which results from the disruption of phosphate excretion and renal ultrastructural damage, also increases odds for adverse renal and cardiovascular outcomes (Block et al., 2019; Isakova, 2011), and in dialysis patients, elevated circulating FGF23 is also an independent risk factor for mortality (Ganz et al., 2019; Isakova, 2011). Therefore, the development of therapies that modify pathways controlling FGF23 in CKD could lead to improved patient outcomes.

A primary manifestation of end‐stage renal disease is the loss of proper iron utilization. In addition to the lack of sufficient erythropoietin (EPO) necessary for the production of red blood cells (Babitt & Lin, 2010; Feldman et al., 2002; Gafter‐Gvili et al., 2019), contributing factors for anemia and iron deficiency in CKD include poor diet, inflammation, compromised iron absorption, bleeding, and EPO resistance (Stauffer & Fan, 2014). During studies defining the molecular mechanisms of ADHR (White et al., 2000), an anemia‐FGF23 linkage was discovered through the study of mice harboring an orthologous point mutation in the *Fgf23* inactivating protease cleavage site. Severe hypophosphatemia was observed in ADHR mice fed a low iron diet to establish iron deficiency anemia (IDA) (Farrow et al., 2011). The IDA increased bone Fgf23 mRNA (Clinkenbeard et al., 2014), resulting in elevated serum intact FGF23. Additionally, anemia and iron deficiency are strong inducers of EPO, and transgenic mice overexpressing EPO were found to have elevated FGF23 (Daryadel et al., 2018). Therefore, overlapping mechanisms of phosphate and iron handling exist, and dysregulation of these pathways can be associated with poor clinical outcomes (Wheeler & Clinkenbeard, 2019).

Ferric citrate (FC, brand name Auryxia®) is a novel class of iron‐based phosphate binder. This agent treats critical CKD manifestations on two therapeutic fronts: binding phosphate in the intestine to lower phosphate absorption and providing iron to treat iron deficiency (Chertow et al., 2017; Ganz et al., 2019). FC has two FDA‐approved uses in the United States: (1) In CKD patients undergoing dialysis, FC is an approved phosphate binder; and (2) in CKD patients who are not currently undergoing dialysis, FC may be used to treat IDA (Ganz et al., 2019). The proposed mechanism for FC’s treatment for iron deficiency arises from its iron backbone. In this regard, unbound FC in the small intestine is absorbed in the ferrous state, making iron available for erythropoiesis (Ganz et al., 2019). Clinical (Block et al., 2019; Maruyama et al., 2018; Yokoyama et al., 2019) and translational (Francis et al., 2019) studies have shown decreases in FGF23 with FC treatment. FGF23 can be reduced in anemic patients by i.v. iron therapy (Wolf et al., 2020), and by FC in rodent models of CKD (Francis et al., 2019); however, it is unknown which manifestations of CKD may be iron‐ or FGF23‐dependent. Further, clinical trial evidence supports that FC can alleviate both iron‐ and phosphate‐related pathologies (Wolf et al., 2020), and since FGF23 is influenced by factors that alter iron handling, the impact of FC on FGF23‐mediated effects are not completely understood. Herein, we used an adenine‐induced CKD mouse model in conjunction with a conditional approach to specifically delete *Fgf23* from osteocytes to isolate FC‐ and FGF23‐dependent phenotypes. The results from this work demonstrated that in a mouse model of CKD, FC increased serum iron in vivo, however it did not suppress blood FGF23 concentrations or alter serum phosphate (a major risk factor in CKD patients) over the time course studied. Finally, FC may improve 1,25D metabolism independently of FGF23, potentially providing other modifiable patient outcomes.

## 2 RESULTS

### 2.1 FGF23 deletion from bone during CKD

**FIGURE 1:**
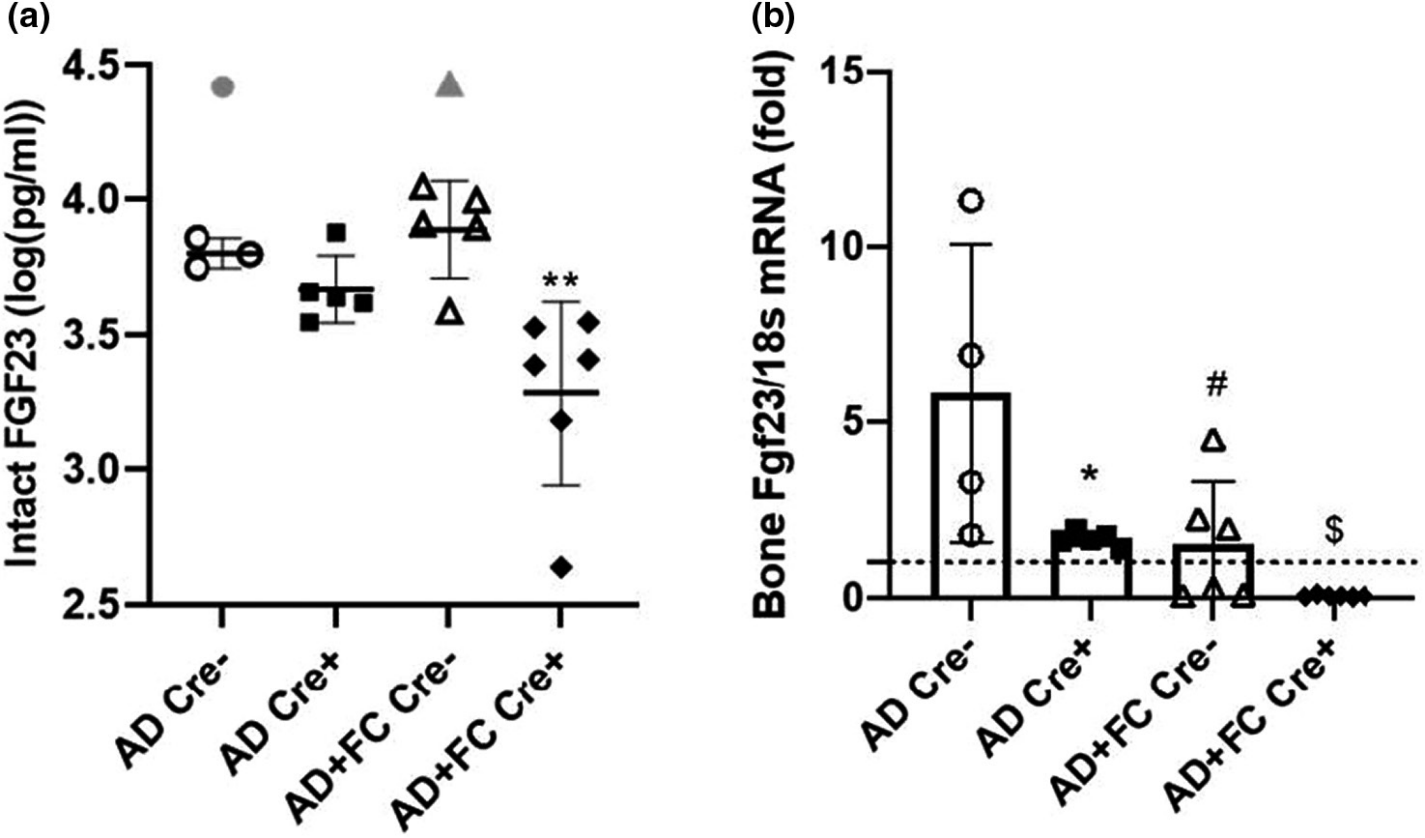
FGF23 with conditional deletion and Ferric citrate (FC) treatment. (a) Post‐treatment intact FGF23 demonstrated significantly lower levels in Cre^+^ mice versus Cre^−^ counterparts; no differences were observed in iFGF23 levels in the FC treated mice versus AD only groups. Three statistical outliers removed. (b) Both Cre^+^ mouse groups showed lower bone Fgf23 mRNA expression compared to Cre^−^ groups and the FC‐treated Cre^−^ group had lower Fgf23 mRNA versus AD‐only controls (*n* = 4–8 mice per group; *p < 0.05; **p < 0.01 versus genotype, same treatment; ^#^ *p* < 0.05 versus treatment, same genotype; ^$^ *p* < 0.05 versus different treatment and genotype via two‐way ANOVA with a Tukey post‐hoc test). Gray points represent data outliers; the dotted line represents relative levels of gene expression in the casein‐diet controls

To determine the roles of FGF23 versus iron handling on CKD phenotypes, a flox‐*Fgf23* allele was targeted for conditional deletion in late‐stage osteoblasts and osteocytes by breeding flox‐Fgf23 conditional mice to the Dmp1‐Cre transgenic line to create flox‐Fgf23/Dmp1‐Cre^+/−^ mice as previously described (Clinkenbeard et al., 2016). An adenine diet (AD; 0.2%), with or without FC (0.5%), was fed to the mice for 6 weeks starting at 8 weeks of age to induce CKD. Serum concentrations of intact FGF23 (iFGF23), the bioactive form of FGF23, were reduced in pre‐treatment samples in the Cre+mice versus Cre‐ (*p* < 0.05), with modest initial differences (*p* < 0.05) between Cre+cohorts (AD/Cre‐: 137.7 ± 9.5 pg/mL; AD/Cre+: 73.1 ± 12.3; AD+FC/Cre‐: 182.5 ± 10.1; and AD FC/Cre+125.9 ± 9.6; *p* < 0.05). Post‐treatment iFGF23 levels were highly elevated compared to pre‐diet samples due to the induction of CKD, in agreement with previous work (Clinkenbeard et al., 2019), and conditional deletion of *Fgf23* also significantly reduced circulating intact iFGF23 post‐treatment (Figure 1a). FC provision, however, did not further influence this reduction in the Cre^+^ mice (Figure 1a), suggesting that circulating bioactive iFGF23 was primarily reduced due to conditional deletion rather than FC‐mediated effects. In bone, Fgf23 mRNA was lower in the Cre^+^ mice, and FC alone had a suppressive effect on Fgf23 mRNA in the Cre^−^ mice during CKD (Figure 1b), however other extra‐osseous sources of FGF23, such as liver, showed no differences across groups (Supplemental Figure 1a). Interestingly, a combination of conditional deletion of osteoblast/osteocyte Fgf23 plus iron administration further suppressed bone Fgf23 mRNA expression (Figure 1b). Body weights were measured every two weeks from the start of dietary treatments and showed that all mouse groups lost weight after 2 weeks, but there were no significant differences between the four groups at the end of the study (Supplemental Figure 1b). Left ventricle hypertrophy (LVH) is a facet of CKD pathogenesis and can be induced by combinatorial effects of inflammation, high circulating FGF23, and progressive vascular calcifications. Thus, heart weight/femur length ratios were tested; however, no significant differences were detected between groups, supporting no effect of FC on heart mass over this time course in CKD animals (Supplemental Figure 1c).

### 2.2 Serum biochemical profile with CKD and FC treatment

Since FGF23 responds to changes in iron utilization, serum biochemical analysis was performed on blood samples collected prior to adenine diet (AD) and at the end of the study. Blood urea nitrogen (BUN), a parameter inversely related to renal function, increased in all groups versus pre‐diet values, demonstrating that the CKD disease phenotype was induced (Table 1). The Cre^+^ mice had higher BUN regardless of whether they received FC, and had elevated serum phosphate concentrations prior to diet administration. After CKD onset, serum phosphate increased in all mouse groups, with an additional increase in Cre^+^ mice versus genotype controls (Table 1). This suggests that FGF23 had a greater effect on phosphate changes when compared with FC in our CKD mouse model. With adenine administration, there was an expected onset of hypoferremia (Table 1), as previously reported (Clinkenbeard et al., 2019). In contrast, regardless of Cre genotype, the mice receiving AD+FC had higher serum iron versus the AD‐only mouse groups. It is not clear why serum iron was lower with Dmp1‐cre mediated reductions of FGF23, however other systemic effects such as the increase in serum phosphate may damage kidney and liver, leading to secondary effects on iron utilization. Serum calcium decreased with diet in all groups, and serum alkaline phosphatase was unchanged regardless of genotype or treatment. Collectively, these biochemistries suggested an FGF23‐mediated effect on serum BUN and phosphate, as these levels were higher in the Cre+mice that had reduced circulating iFGF23. In contrast, FC treatment was associated with increased serum iron which was not influenced by iFGF23.

**TABLE 1:** Serum Biochemical analysis

	AD Cre−		^AD Cre+^		AD+FC Cre−		AD+FC Cre+	
Serum biochemistry	Pre	Post	Pre	Post	Pre	Post	Pre	Post
Serum phosphorous	7.39 ± 0.90	11.90 ± 3.17^e^	10.27 ± 0.49^b^	16.66 ± 1.63^a^, ^f^	7.63 ± 0.54	11.95 ± 1.44^e^	10.62 ± 0.55^b^	15.34 ± 0.9^f^
Total serum iron	39.00 ± 1.35	21.18 ± 3.43^e^	39.25 ± 1.99	14.30 ± 1.84^a^, ^e^, ^f^	31.83 ± 2.57^d^	25.33 ± 1.78^c^	31.58 ± 1.14^d^	22.4 ± 0.8^d^, ^f^
BUN	21.10 ± 0.99	96.13 ± 25.55^f^	24.82 ± 0.87	135.50 ± 10.7^b^, ^f^	22.44 ± 2.52	90.41 ± 5.33^f^	18.79 ± 1.21	126.2 ± 4.1^b^, ^f^
Alk phos	127.50 ± 10.31	139.50 ± 21.72	107.50 ± 5.44	110.00 ± 21.48	110.83 ± 4.17	106.50 ± 3.69	108.33 ± 10.22	113.00 ± 15.4
Calcium	7.53 ± 0.72	5.85 ± 0.84	8.77 ± 0.62	5.32 ± 0.26^e^	9.55 ± 0.44 ^b^	5.78 ± 0.69^f^	9.55 ± 0.44	5.31 ± 0.6^f^

Note

Biochemical analysis showing pre‐treatment at 8 weeks of age and post‐treatment after 6 weeks of adenine diet (AD), or AD combined with FC (AD+FC); *n* = 4–8 mice per group.

^a^ *p* < 0.05.

^b^ *p* < 0.01 versus genotype, same treatment.

^c^ *p* < 0.05.

^d^ *p* < 0.01 versus treatment, same genotype via two‐way ANOVA with a Tukey post‐hoc test.

^e^ *p* < 0.05.

^f^ *p* < 0.01 pre‐ versus post‐diet with the same treatment and genotype via student's *t*‐Test.

### 2.3 Effects of FC on vitamin D regulating enzymes

**FIGURE 2:**
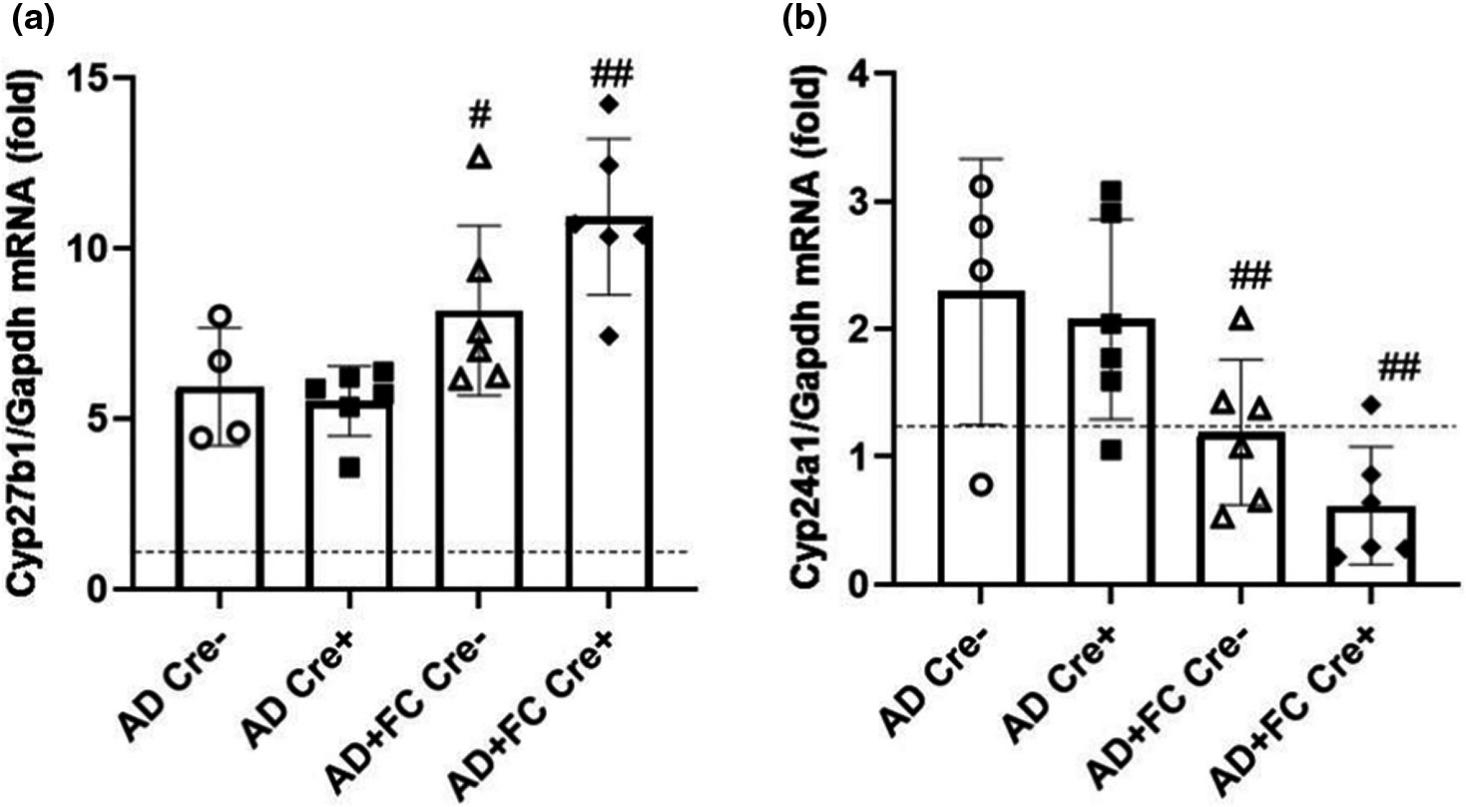
Vitamin D metabolic genes in chronic kidney disease. (a) Kidney Cyp27b1 mRNA levels were higher in the mice fed Ferric citrate (FC) regardless of genotype. (b) Kidney Cyp24a1 mRNA levels were lower in the mice on FC regardless of genotype. (*n* = 4–8 mice per group; ^#^ *p* < 0.05, ^##^ *p* < 0.01 versus treatment, same genotype via two‐way ANOVA with a Tukey post‐hoc test). Dotted line represents relative levels of gene expression in casein‐diet controls

During CKD disease progression, normal blood phosphate can be maintained by increased circulating iFGF23 concentrations to act on the failing kidney. However, this elevation increases the catabolic Cyp24a1 expression to lower plasma 1,25D which cannot be rescued by increased anabolic Cyp27b1. The mice receiving AD+FC had significantly higher renal Cyp27b1 mRNA expression than those receiving AD‐only (Figure 2a), and reductions in plasma FGF23 due to the presence of Dmp1‐cre were not associated with reduced Cyp27b1. This most likely occurred since circulating intact FGF23 is still markedly higher than in normal mice. Interestingly, the mice receiving AD+FC, regardless of genotype, showed lower Cyp24a1 expression than AD‐only diet groups (Figure 2b). These results suggest that FC may partially protect 1,25D metabolic enzyme expression during CKD, and this effect is independent of the genetically‐reduced circulating FGF23 concentrations.

### 2.4 Inflammation and fibrosis responses with FC treatment

**FIGURE 3:**
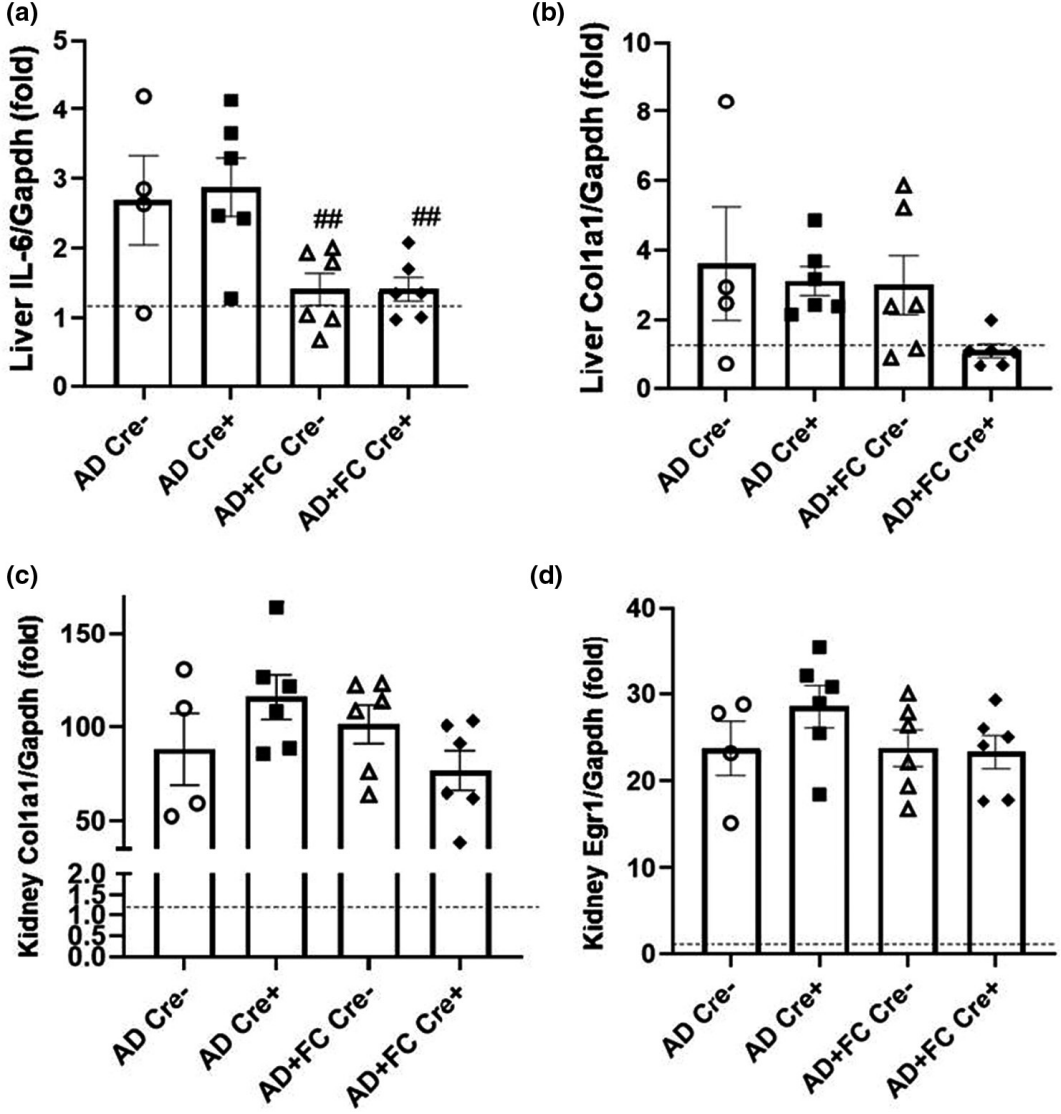
Markers of inflammation and fibrosis. (a) Liver IL‐6 mRNA levels were lower in the mice fed Ferric citrate than those receiving AD only. (b) Liver Col1a1 mRNA levels, (c) kidney Col1a1 mRNA levels, and (d) kidney Egr1 mRNA expression, were not different across groups. (*n* = 4–8 mice per group; ^#^ *p* < 0.05, ^##^ *p* < 0.01 versus treatment, same genotype via two‐way ANOVA with a Tukey post‐hoc test). Dotted line represents relative levels of gene expression in the casein‐diet controls

CKD is a multifactorial disease with a hallmark development of persistent inflammation, which can be associated with marked fibrosis, and poses a negative impact on CKD progression. Hepatic expression of the inflammatory cytokine IL‐6 mRNA was significantly lower in both the flox‐Fgf23/Dmp1‐Cre^+/−^ groups treated with AD + FC compared to those receiving AD‐only (Figure 3a), suggesting that FC may be protective for upregulation of IL‐6 mRNA. The fibrosis marker Type 1 Collagen (Col1a1) mRNA expression was upregulated in liver versus casein‐diet controls (shown as a dotted line), but no difference was observed between genotypes or FC administration (Figure 3b). In kidney, Col1a1 (Figure 3c) and a stress response gene Early growth response gene‐1 (Egr1) mRNAs (Figure 3d) were approximately 100‐ and 20‐fold elevated versus casein diet control mice (shown as a dotted line), respectively. However, there were no significant differences across groups, suggesting that the fibrosis associated with the adenine CKD mouse model over the six‐week time course was independent of FC and FGF23.

### 2.5 Molecular analyses of iron utilization

**FIGURE 4:**
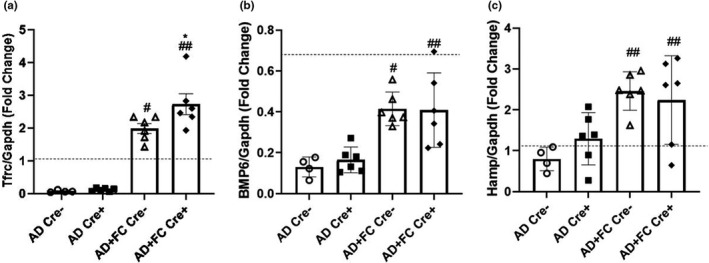
Genes controlling iron utilization. (a) Liver Tfrc mRNA expression was higher in the mice receiving Ferric citrate (FC) regardless of genotype, and Tfrc mRNA in Cre^+^ mice fed FC was higher than the Cre^−^ control. (b) Liver Bmp6 mRNA levels were higher in the mice treated with FC regardless of genotype. (c) Liver Hamp mRNA was elevated in the mice fed FC regardless of genotype. (*n* = 4–8 mice per group; *p < 0.05 versus genotype, same treatment; ^#^ *p* < 0.05; ^##^ *p* < 0.01 versus treatment, same genotype via two‐way ANOVA with a Tukey post‐hoc test). Dotted line represents relative levels of gene expression in the casein‐diet controls

Using the conditional deletion of *Fgf23* from bone to lower circulating iFGF23 provided a unique opportunity to differentiate between FGF23‐ and FC‐mediated changes in genes controlling the physiological response to iron. The AD‐only groups almost completely suppressed liver Transferrin receptor (Tfrc) mRNA versus normal controls, and the flox‐Fgf23/Dmp1‐Cre^+/−^ mouse groups receiving AD+FC increased expression of liver Tfrc mRNA expression versus the AD‐only groups (Figure 4a). Unexpectedly, the flox‐Fgf23/Dmp1‐Cre^+^ mice receiving AD+FC had additional increases in Tfrc expression versus AD alone (Figure 4a). Whether this finding is due to the lower concentration of FC used versus previous studies and thus more modest increases in serum levels, or is specific for the AD‐CKD model is currently unknown. Additionally, liver Bone morphogenetic protein 6 (Bmp6) mRNA levels were suppressed versus normal mice and significantly improved in the AD+FC groups versus AD‐only, regardless of Fgf23 expression (Figure 4b). Finally, liver hepcidin (Hamp) mRNA levels were elevated in the AD+FC groups, regardless of circulating iFGF23 (Figure 4c), consistent with the Bmp6 and Tfrc mRNA increases. Collectively, these results support an FC‐mediated effect on liver iron utilization and storage, with potential enhancement of the Tfrc effects during the conditional reduction in circulating iFGF23.

### 2.6 Bone microarchitecture and mineral matrix properties

**FIGURE 5:**
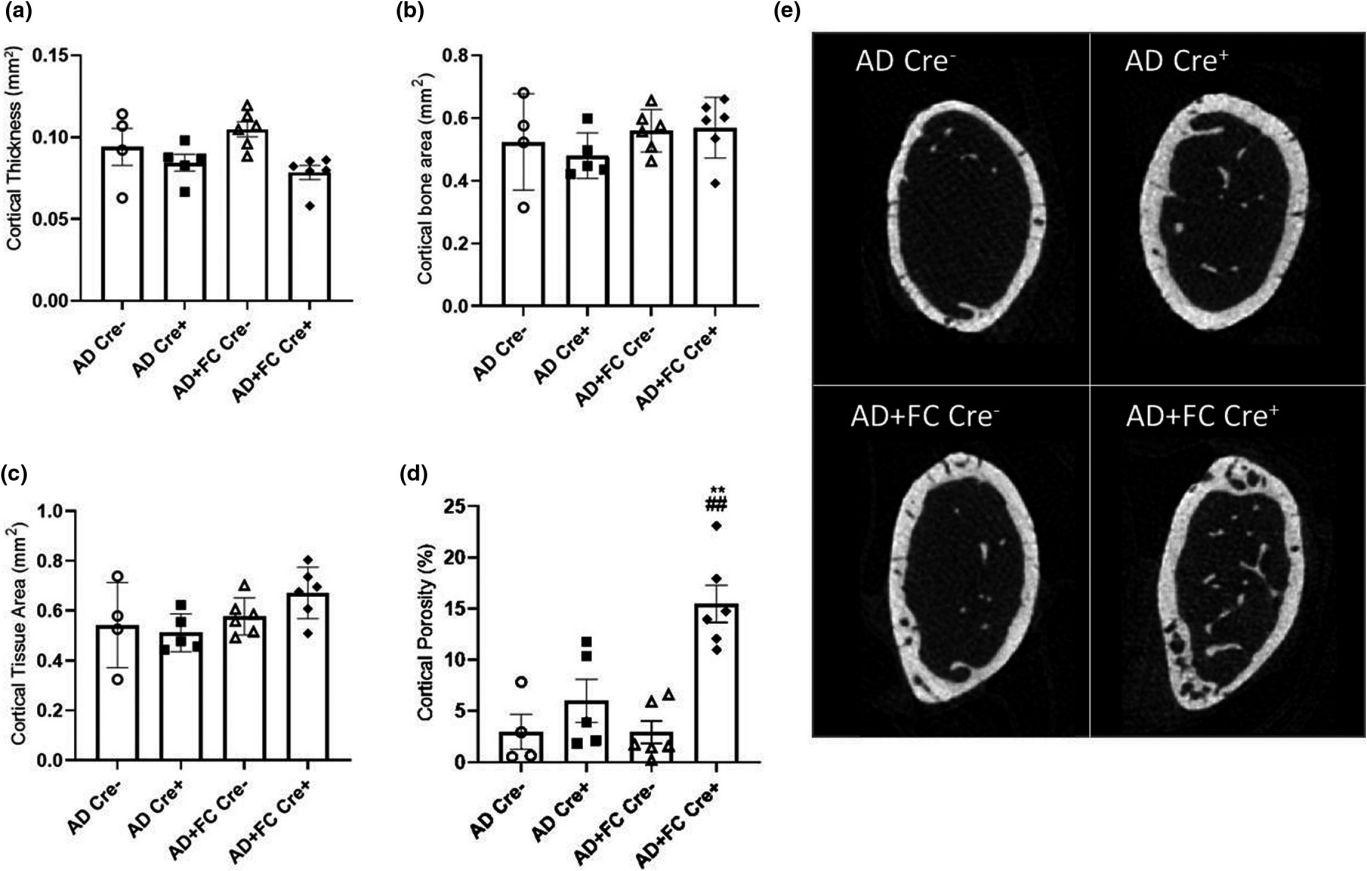
Cortical bone structure. (a) Cortical thickness, (b) cortical bone area, and (c) cortical tissue area were not different between groups. (d) Cortical porosity was higher in the Cre^+^ Ferric citrate (FC)‐treated mice. (e) The median µCT image for each group is shown. (*n* = 4−8 mice per group; **p < 0.01 versus genotype, same treatment; ^#^ *p* < 0.05, ^##^ *p* < 0.01 versus treatment, same genotype via two‐way ANOVA with a Tukey post‐hoc test)

In animal models of CKD and in patients, PTH elevation drives osteoclast activation resulting in cortical porosity, leading to an increased risk of skeletal fractures (McNerny & Nickolas, 2017; Nickolas et al., 2013). Disturbances in iron utilization (from chronic iron loading or severe anemia) may also negatively affect bone structure. The effects of genetic reductions in FGF23 with and without FC treatment on CKD bone properties were thus examined by μCT. Femur cortical thickness (Figure 5a), cortical bone area (Figure 5b), and cortical tissue area (Figure 5c) were not significantly different between groups. In contrast, the Cre^+^ mice fed the combined AD+FC diet showed higher cortical porosity than other groups (*p* < 0.001; Figure 5d; the median µCT image for each group is shown in Figure 5e). Analysis of femur trabecular bone showed no differences in trabecular bone volume/tissue volume, trabecular number, separation, or thickness between groups (Supplemental Table 1). To further assess bone at the matrix level, Raman spectroscopy was performed on femur cortical bone. There were no differences in matrix properties, including mineral crystallinity/maturity, type B carbonate substitution, or mineral‐to‐matrix ratio between groups (Supplemental Data, Table 2). Collectively, these data support that FGF23 may provide protective effects for bone with changes in iron utilization during CKD, or that the combination of supraphysiological serum phosphate plus an increase of serum iron altered skeletal properties, and that FC did not significantly influence mineral or matrix quality.

In summary, using a conditional deletion approach for Fgf23, effects of FGF23 and FC were differentiated during modeled CKD. These studies showed that BUN and serum phosphate concentrations were FGF23‐dependent parameters. Regardless of FGF23 status, the provision of FC corrected the prevailing hypoferremia, as well as normalized hallmark genes associated with controlling iron utilization, and influenced vitamin D metabolic enzyme expression. Finally, FC treatment during CKD had no detrimental effects on skeletal mineral matrix composition or maturity, and FC treatment in the absence of FGF23 revealed potential FGF23‐protective actions on bone.

## 3 DISCUSSION

CKD is a major global health issue with high prevalence. With the loss of renal function, patients can have severe disturbances in mineral metabolism, dysregulated endocrine function, inflammation, anemia, and tissue fibrosis. FC emerged as a promising therapy for CKD patients due to its dual effects on iron‐ and phosphate‐related pathologies. To lower phosphate absorption, FC binds phosphate in the gastrointestinal tract to form ferric phosphorous, which is insoluble and can be excreted (Ganz et al., 2019) and raises total serum iron due to its iron molecular backbone (Ganz et al., 2019; Lewis et al., 2015). In this study, we focused on the impact of iron supplementation by FC and FGF23‐mediated phosphate regulation to investigate how these would affect CKD progression and outcomes.

Since circulating FGF23 is increased by anemia (Farrow et al., 2011; Imel et al., 2011; Nam et al., 2018) and lowered by iron repletion (Imel et al., 2020), in our experiments, a targeted deletion of FGF23 from osteoblasts/osteocytes was used in concert with FC treatment to separate FGF23‐ versus FC‐driven effects. As expected, the Cre^+^ mice with bone‐specific *Fgf23* deletion had lower serum iFGF23 levels versus Cre^−^ mice. Clinical trials have shown that circulating FGF23 was reduced by FC treatment, consistent with studies in a genetic mouse model of CKD, where FGF23 was lowered by high dose FC (Block et al., 2015, 2019; Francis et al., 2019). In the experiments herein, this effect was not observed over the time course tested. In contrast to previous work, (Francis et al., 2019) our studies used a dose of 0.5% added FC. In the reported studies, higher concentrations of 5% dietary FC were associated with reductions in FGF23 and serum phosphorous, as well as increased serum iron (Francis et al., 2019). In the present study, the mice with lower iFGF23 due to conditional deletion had higher serum BUN, consistent with previous findings (Clinkenbeard et al., 2019). No differences in serum BUN were observed in the FC‐treated groups, compared to those receiving AD alone, which is consistent with lowered FGF23 being the primary driver of this phenotype (Francis et al., 2019). Although FC is known to reduce serum phosphate in patients with CKD and at higher doses in mice, we did not observe this effect. Following *Fgf23* deletion during CKD, serum phosphorous was elevated, as observed in previous studies, however, FC had no effect on this parameter. Perhaps with a longer treatment course and/or higher doses, an effect of FC may be realized. FGF23 is known to be markedly elevated in late‐stage CKD, and it was demonstrated that normalization of iron utilization in mice with CKD via increasing EPO directly or indirectly resulted in lowered FGF23 (Noonan et al., 2020, 2021). The delivery of FC reversed the hypoferremia associated with the adenine‐CKD diet in the presence or absence of bone Fgf23. This effect is consistent with the known response of patients with CKD to FC, which also resulted in improved hemoglobin and hematocrit in clinical trials (Block et al., 2019; Maruyama et al., 2018). Thus, although FGF23 has been implicated in iron handling as demonstrated by the delivery of FGF23 inhibitory peptides during CKD (Agoro et al., 2018), the circulating FGF23 does not appear to be involved in the mechanisms whereby FC normalizes blood iron concentrations.

The FGF23‐mediated effect of suppressing renal 1,25D production during CKD through activation of Cyp24a1 can have severe downstream endocrine effects, including causing hyperparathyroidism and metabolic bone disease. In attempting to distinguish FGF23‐ and FC‐specific mechanisms, interestingly, an increase in anabolic Cyp27b1 mRNA and a reduction in the catabolic Cyp24a1 was associated with FC treatment and was not affected by the changes in FGF23 due to conditional deletion from bone. These results showed a similar effect observed in a Col4a3‐KO CKD mouse model, however, the extent to which FGF23 was involved in this process remains unknown (Francis et al., 2019). Importantly, both Cyp27b1 and Cyp24a1 are heme‐containing enzymes, therefore it is possible that the exogenous iron provided from the FC iron backbone in FC may influence the expression of these enzymes (Jones et al., 2014). To understand the molecular nature of these effects, future studies could examine the local roles of iron utilization in proximal tubule cells in isolation as well as the assessment of plasma 1,25D levels to determine whether changes in the vitamin D metabolic enzyme expression coincided with changes in circulating 1,25D concentrations.

Strengths of the present work include that the adenine model of CKD recapitulates the anemia and many of the downstream manifestations observed in CKD patients. In this regard, we and others previously demonstrated that this model has hypoferremia, as well as reduced hematocrit and hemoglobin, tissue fibrosis, and increased markers of inflammation (Clinkenbeard et al., 2019; Noonan et al., 2020). Previous clinical studies in humans (Wolf et al., 2013) and translational experiments in mice (Farrow et al., 2011), as well as isolated osteoblast/osteocyte cells (Noonan et al., 2021), have also shown that FGF23 is driven by anemia/hypoxia (Clinkenbeard et al., 2019). Studying mice with CKD under dual conditions of treatment with exogenous iron on a genetic background of conditional *Fgf23* deletion was another strength of this work, providing a unique opportunity to dissect phenotypes downstream of anemia. In the current study, FC treatment was associated with elevated liver Tfrc (transferrin receptor) mRNA. In contrast, a previous study showed that in mice with CKD, higher doses of FC lowered liver Tfrc mRNA (Francis et al., 2019). Under the dosage and time point used in the experiments performed herein, the transport of iron into tissues may still be in process, whereas in situations of higher FC doses, iron saturation could reduce Tfrc to protect tissues from iron overload. Future studies could measure iron levels in liver and kidney to test these correlations. Consistent with earlier reports (Francis et al., 2019), liver hepcidin (Hamp) mRNA was elevated in mice receiving FC. Hepcidin lowers intestinal iron absorption, and can be regulated by both BMP6 (an iron homeostatic factor) and IL‐6 (an inflammatory cytokine). We found that in FC‐treated mice, liver Bmp6 mRNA expression was increased, whereas IL‐6 mRNA decreased, therefore our study supports that in the adenine model, FC may have activated hepcidin via BMP6 rather than downstream of IL‐6 mediated inflammatory pathways, and that these outcomes were independent of FGF23. The exact mechanisms for the increased IL‐6 levels are not known, but could be important for understanding translational aspects of FC treatment in CKD patients with marked inflammation.

The bone disease associated with CKD can be severe and in part, results from reduced 1,25D which increases PTH and its stimulatory actions on osteoclast‐mediated bone resorption. Although we were unable to measure all endocrine variables due to small sample volumes obtained during the severe CKD induced by adenine, including PTH, the current study found that there was a trend towards increased porosity with reductions of iFGF23 due to conditional *Fgf23* deletion. The FC treated group on the *Fgf23* conditional‐null background had significantly higher porosity, perhaps suggesting a protective role for FGF23 on bone during improved iron utilization. Indeed, with increased circulating iron, if FGF23 has effects on reducing local or systemic oxidative stress (Czaya & Faul, 2019) then a suppression of FGF23 to levels that would elevate serum phosphate during CKD may further negatively influence bone properties. Additionally, whether higher iron concentrations in the context of hyperphosphatemia had direct effects on the skeleton remains unknown. To further examine the skeletal CKD phenotypes, Raman spectroscopy was performed. These analyses found no differences across the groups, supporting that over the time course, FC did not have detrimental effects on tissue‐level matrix composition or mineral crystallinity in cortical bone. Future studies could focus upon whether the influence of FGF23 on bone during changes in iron handling were due to local or systemic effects.

There are several limitations to our studies, including that iFGF23 is not fully suppressed with conditional targeting of this hormone in late osteoblasts/osteocytes, and circulating concentrations during CKD onset and progression remained well above normal mouse serum levels. Although we found no differences in Fgf23 mRNA levels in liver, another source of FGF23 (Agoro et al., 2021; White et al., 2000), potentially marrow (Clinkenbeard et al., 2017) or spleen (Bansal et al., 2017) could secrete enough FGF23 during CKD to attempt to maintain serum levels. Additionally, partial efficiency of the Dmp1‐cre or the production of FGF23 from other bone derived cells cannot be ruled out at this time. Importantly, serum phosphate was increased in the Cre^+^ mice, therefore, the level of Fgf23 reductions in bone in these groups of mice had a significant effect on systemic mineral metabolism. Further, as described above, anemia has been recognized as a very strong stimulator of Fgf23 mRNA levels in bone in vivo (Farrow et al., 2011) and in cells (Noonan et al., 2021), and serum phosphate also drives Fgf23 production (Saito et al., 2005). Considering the marked hypoferremia and hyperphosphatemia observed in the conditional‐null mice, although the Dmp1‐Cre is known to efficiently recombine the flox‐Fgf23 allele (Clinkenbeard et al., 2016), the sum of both of these systemic Fgf23 drivers may override the suppressive abilities of the targeted deletion. Acute correction of anemia with EPO and new agents that increase endogenous EPO production, the hypoxia‐inducible factor‐prolylhydroxylase inhibitors (HIF‐PHI), have been shown to fully rescue complete blood counts in the adenine‐CKD model (Noonan et al., 2020, 2021). However, in a similar manner, iFGF23 is not completely suppressed following the administration of these agents. Thus, FGF23 stimulation may be due to other biochemical or local factors.

In summary, this study performed targeted deletion of *Fgf23* in combination with FC treatment to dissect the roles of FGF23 and iron on key CKD manifestations. The present work revealed distinct FGF23‐mediated effects, such as controlling serum phosphate and BUN, which were both increased with FGF23 deletion. Independent actions of FC included increased serum iron, with improved iron utilization parameters and reduced markers of inflammation.

## 4 MATERIALS AND METHODS

### 4.1 Animal studies

Animal studies were approved by and performed according to the Institutional Animal Care and Use Committee (IACUC) for the Indiana University School of Medicine, and comply with the NIH guidelines for the use of animals in research. All mice used in this study were male, and derived in house from Floxed‐Fgf23/Dmp1‐Cre mice as previously described (Clinkenbeard et al., 2016). The casein control mice used were C57BL/6 (C57) purchased from Jackson Labs. Mice were euthanized by CO_2_ inhalation followed with cervical dislocation, and blood was collected by cardiac puncture for serum and plasma (collected in EDTA tubes). Where indicated, facial vein bleeds were used for measures of plasma intact FGF23 concentrations.

### 4.2 Rodent diets

All mice received a normal rodent diet from the IUSM animal facility (2018SX, Harlan Teklad) until placed on experimental diets. At 8 weeks of age mice were provided a 0.2% adenine containing diet (TD.160020; Envigo) for 6 weeks, or a 0.2% adenine containing diet supplemented with 0.5% Ferric Citrate (provided by Akebia Therapeutics). Control mice were switch to Casein diet (TD.150303; Envigo) for 6 weeks at 8 weeks of age. Diets and water were provided ad libitum throughout the study.

### 4.3 Serum biochemistries

Blood samples were collected from mice for interim analyses by facial vein bleed and at the time of euthanasia by cardiac puncture according to approved protocols. Where indicated, mice were facial vein bled for interim analysis, collecting less than 5% of the total blood volume to mitigate potential effects on the parameters tested (Diehl et al., 2001). Routine serum biochemistries were determined in the Laboratory of the Clinical and Translational Sciences Institute (CTSI) of the Indiana University School of Medicine using an automated COBAS MIRA Plus Chemistry Analyzer (Roche Diagnostics; Indianapolis, IN).

### 4.4 Hormone ELISAs

Plasma “intact” FGF23 (iFGF23) concentrations were assessed using commercial ELISAs specific for mouse/rat (Quidel, Inc., San Diego, CA). All experiments were performed according to manufacturer's protocol.

### 4.5 RNA preparation

Kidney, liver, and long bones (flushed of marrow) were harvested and homogenized in 1 mL of Trizol reagent (Invitrogen/Life Technologies, Inc.; Grand Island, NY) according to the manufacturer’s protocol using a Bullet Blender (Next Advance, Inc.; Troy, NY), then further purified using the RNeasy Kit (Qiagen, Inc.; Germantown, MD).

### 4.6 Quantitative RT‐PCR (qPCR)

Mouse *18S* or *Gapdh* was used as an internal control by RT‐qPCR. The qPCR primers and probes of *Gapdh*, *Egr1*, *Cyp27b1*, *Cyp24a1*, *Hamp*, *Bmp6*, *Tfrc*, *Col1a1*, *IL6*, *Fgf23*, and *18S* were purchased as pre‐optimized reagents (Applied Biosystems/Life Technologies, Inc.) and the TaqMan One‐Step RT‐PCR kit was used to perform qPCR. PCR conditions for all experiments were: 30 min 48°C, 10 min 95°C, followed by 40 cycles of 15 s 95°C and 1 min 60°C. The data was collected and analyzed by a StepOne Plus system (Applied Biosystems/Life Technologies, Inc.).

### 4.7 Miro‐computed tomography

Femurs were group‐scanned (3 per scan) at 8µm voxel size with 0.5 aluminum filter, 2 frame averaging, and 0.7 rotation step on a Skyscan 1172 (Bruker, Billerica, MA, USA). Trabecular bone properties were measured in a 1 mm region located proximal to the growth plate in the distal femur. Cortical bone was analyzed in 5 consecutive slices 2.5 mm’s proximal to the fusion of the distal femur growth plate.

### 4.8 Raman spectroscopy

The anterior surfaces of mouse femora were sanded with silicon carbide sandpaper and polished with a 3 and 0.05 µm diamond suspension to create a flat, smooth surface for Raman spectroscopy. Raman spectra were acquired at eight locations per femur with an 8 s exposure and eight accumulations. Spectral data underwent baseline removal with WiRE intelligent fitting and cosmic rays were removed (Renishaw, Wotton‐under‐Edge, UK). Spectra were smoothed with a 3rd order Savitsky‐Golay filter across nine points. Mineral‐to‐matrix ratio (ν_1_PO_4_^3−^/Amide I band areas), type B carbonate substitution (ν_1_CO_3_^2−^/ν_1_PO_4_^3−^ band areas), and mineral crystallinity/maturity (inverse of full width at half maximum of ν_1_PO_4_^3−^) were calculated using a custom MATLAB script. Final parameters were averaged across the eight measured locations, resulting in one value per parameter per femur.

### 4.9 Statistical analysis

Statistical analysis of the data was performed by two‐way ANOVA followed by a Tukey post‐hoc test, or a Student’s *t*‐test where appropriate. Data outliers were determined by performing Grubb’s test and removed. Significance for all tests was set at *p* < 0.05. Data in bar graphs are presented as means ± standard error of the mean (SEM).

## CONFLICT OF INTEREST

KEW receives royalties for licensing the *FGF23* gene to Kyowa Hakko Kirin, Ltd, and receives funding from Akebia Therapeutics, Inc and Calico Labs.

## AUTHORS CONTRIBUTIONS

Michael P. Liesen, Pu Ni, Megan L. Noonan and Kenneth E. White designed the study and wrote the manuscript. Michael P. Liesen, Pu Ni, Erica L. Clinkenbeard, Elizabeth A. Swallow, John G. Damrath, Joseph M. Wallace, Matthew R. Allen, Rafiou Agoro, Julia M. Hum, and Megan L. Noonan performed experiments, and/or analyzed the data. All authors reviewed and edited the final version of the manuscript.

## Supporting information



Supplementary Information
