# Supplementary material for: Segregating the effects of ferric citrate‐mediated iron utilization and FGF23 in a mouse model of CKD
Source: Physiol Rep. 2022 Jun 3;10(11):e15307. doi: 10.14814/phy2.15307 (PMC9163801; doi:10.14814/phy2.15307)
Supplement: Supplementary file 1 — Supplementary Information [file PHY2-10-e15307-s001.docx]

**Supplemental data**

**
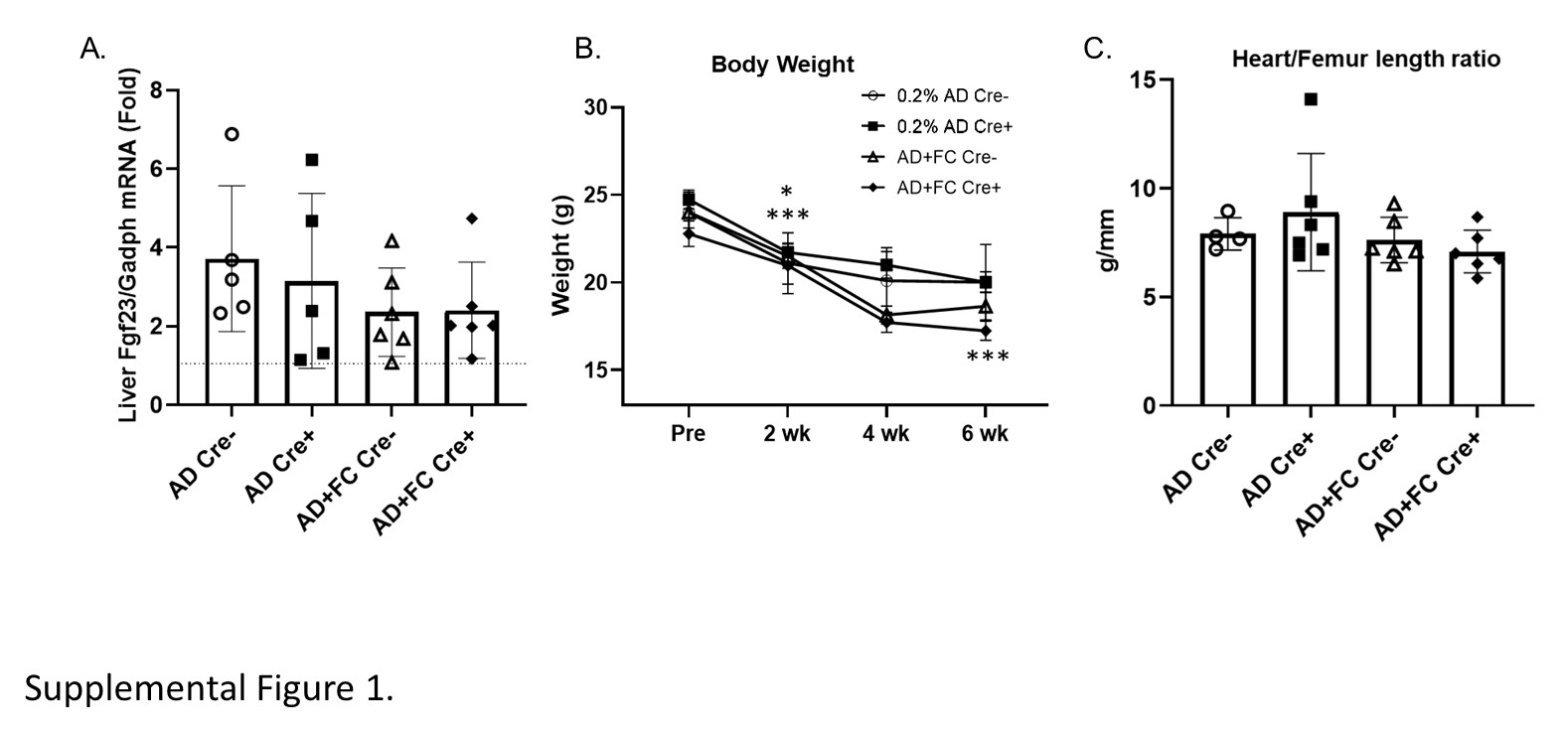
**

**Supplemental Figure 1. Gross manifestations of CKD.** (A) Liver Fgf23 mRNA was measured by qPCR as an alternative source of FGF23 but was not different between groups. (B) Mouse cohorts lost body weight from the start of the adenine diet to 2 weeks (‘wks’) (AD Cre-: p<0.05; AD Cre+: p<0.001; AD+FC Cre-: p<0.01; AD+FC Cre+: p<0.05) and by the end of the study (AD Cre-: NS; AD Cre+: p<0.001; AD+FC Cre-: p<0.001; AD+FC Cre+: p<0.001), however there were no significant differences between groups (*p<0.05, **p<0.01, ***p<0.001). (B) Heart weight/femur length ratios showed no significant changes among mouse groups. (n=4-8 mice per group).

|  | **AD Cre-** | **AD Cre+** | **AD+FC Cre-** | **AD+FC Cre+** |
| --- | --- | --- | --- | --- |
| Trabecular BV/TV (%) | 5.95 ± 1.49 | 5.69 ± 0.54 | 5.78 ± 0.44 | 9.29 ± 1.60 |
| Trabecular thickness (mm) | 0.04 ± 0.004 | 0.04 ± 0.001 | 0.04 ± 0.01 | 0.04 ± 0.003 |
| Trabecular separation (mm) | 0.26 ± 0.02 | 0.26 ± 0.006 | 0.26 ± 0.01 | 0.22 ± 0.01 |
| Trabecular number (1/mm) | 1.55 ± 0.24 | 1.55 ± 0.10 | 1.55 ± 0.11 | 2.13 ± 0.22 |

Supplemental Table 1. Trabecular bone analysis. μCT of the trabecular bone showed no change across groups in percent bone volume, trabecular thickness, trabecular separation, or trabecular number. (n=4-8 mice per group). BV/TV: bone volume/tissue volume.

|  | **AD Cre-** | **AD Cre+** | **AD+FC Cre-** | **AD+FC Cre+** |
| --- | --- | --- | --- | --- |
| **ν_1_CO_3_^2-^/ν_1_PO_4_^3-^** | 0.1581 ± 0.0035 | 0.1588 ± 0.0029 | 0.1638 ± 0.0022 | 0.1592 ± 0.0024 |
| **v1CO3/v1PO4 Intensity** | 0.1387 ± 0.0002 | 0.1358 ± 0.0034 | 0.1392 ± 0.0017 | 0.1315 ± 0.0020 |
| **Mineral Crystallinity/Maturity (1/FWHM of ν_1_PO_4_^3-^)** | 0.0591 ± 0.0012 | 0.0596 ± 0.0013 | 0.0585 ± 0.0006 | 0.0601 ± 0.0003 |
| **ν_1_PO_4_^3-^/Amide I** | 8.56 ± 0.76 | 7.83 ± 0.56 | 9.17 ± 0.63 | 7.62 ± 0.38 |

Supplemental Table 2. Raman spectroscopy analysis of femur. There were no statistical differences in matrix properties across groups irrespective of conditional deletion of FGF23 with or without FC treatment. FWHM = full width at half maximum.
